# Weight-aware semi-supervised self-ensembling framework for interior decoration style classification

**DOI:** 10.3389/frai.2025.1645877

**Published:** 2025-09-09

**Authors:** Lichun Guo, Hao Zeng, Junliang Wang, Shuang Liang, Wenlong Hang

**Affiliations:** ^1^College of Art and Design, Nanjing Audit University Jinshen College, Nanjing, China; ^2^College of Computer and Information Engineering, Nanjing Tech University, Nanjing, China; ^3^School of Internet of Things, Nanjing University of Posts and Telecommunications, Nanjing, China

**Keywords:** self-ensembling, consistency regularization, contrastive learning, interior decoration style, semi-supervised learning

## Abstract

Automatic classification of interior decoration styles has great potential to guide and streamline the design process. Despite recent advancements, it remains challenging to construct an accurate interior decoration style recognition model due to the scarcity of expert annotations. In this article, we develop a new weight-aware semi-supervised self-ensembling framework for interior decoration style recognition, which selectively leverages the abundant unlabeled data to address the aforementioned challenge. Specifically, we devise a weight module that utilizes a truncated Gaussian function to automatically assess the reliability of unlabeled data. This enables more reliable unlabeled samples to be adaptively assigned higher weights during the training process. By incorporating adaptive weights, we devise a weighted consistency regularization to enforce consistent predictions for reliable unlabeled data under different perturbations. Furthermore, we devise a weighted relation consistency regularization to preserve the semantic relationships of reliable unlabeled data across various perturbations. Additionally, we introduce a weighted class-aware contrastive learning regularization to improve the model's discriminative feature learning capability using reliable unlabeled data. The synergistic learning of weighted consistency regularization, weighted relation consistency, and weighted class-aware contrastive learning significantly enhances the model's generalizability. Extensive experiments conducted on interior decoration style image datasets demonstrate the superior performance of our framework compared to existing semi-supervised learning methods.

## 1 Introduction

Automatic classification of interior decoration styles has great potential in guiding and streamlining the design process. By using artificial intelligence techniques, it is possible to analyze various design elements (e.g., color schemes, furniture types, materials, and layouts) to categorize a given interior decoration into predefined categories, including Country, Chinese, European, Simple, and others. This can help designers make informed decisions, provide clients with more personalized recommendations, and speed up the design process (Shan et al., 2022; Guo et al., 2024; Liu et al., 2021). At present, deep learning methods play a pivotal role in interior design practice and are extensively applied across various domains, including interior decoration style classification (Kim and Lee, 2020), style colorization (Tong et al., 2019), style design (Wu et al., 2024), and other related aspects.

Although deep learning methods have achieved remarkable success in various visual recognition tasks, their performance heavily relies on large-scale, high-quality labeled datasets. In the context of interior decoration style classification, obtaining such labeled data is both labor-intensive and time-consuming. In contrast, unlabeled images are easier to collect in real-world scenarios. Semi-supervised learning methods have demonstrated significant potential by effectively exploiting unlabeled data to enhance model performance. Consequently, semi-supervised learning is progressively emerging as the predominant method for interior decoration style classification. Most existing semi-supervised learning methods mainly rely on the smoothness assumption, which posits that nearby samples tend to belong to the same class. Based on this principle, the consistency learning strategy (Tarvainen, 2017) leverages unlabeled data by enforcing consistent outputs from the network when subjected to input or model perturbations. Another strategy is pseudo-labeling (Iscen et al., 2019), where the pseudo-labels are generated for the unlabeled data and then combined with labeled samples to train the model. A prevailing trend is to integrate the consistency learning and pseudo-labeling techniques to enhance the effectiveness of semi-supervised learning (Chen et al., 2021; Liu et al., 2022). To date, semi-supervised learning methods have achieved notable success in various real-world applications, including image recognition (Wu et al., 2022; Hang et al., 2022), image segmentation (Hang et al., 2025, 2020), and text classification (Duarte and Berton, 2023; Han et al., 2023).

Despite promising progress, existing semi-supervised learning methods still encounter major difficulties when the pseudo-labels of unlabeled data are unreliable. For consistency learning-based methods, perturbations applied to inaccurate predictions may result in potentially erroneous training signals, thereby degrading the effectiveness of consistency learning (Liu et al., 2022). Besides, for pseudo-labeling-based semi-supervised learning methods, incorporating inaccurate unlabeled data into training process introduces confirmation bias, which significantly deteriorates model performance (Hang et al., 2024). In light of this, various strategies have been proposed to mitigate the adverse effects of unreliable pseudo-labels, yielding promising results across multiple applications. For pseudo-labeling-based methods, a commonly adopted strategy involves setting a high confidence threshold to filter out low-confidence pseudo-labeled samples (Berthelot et al., 2019; Sohn et al., 2020; Yu et al., 2019). Although effective, the quality of pseudo-labels still heavily depend on the predefined confidence threshold, making the confirmation bias problem caused by unreliable pseudo-labels unresolved. For example, FixMatch (Sohn et al., 2020) adopted a confidence threshold 0.95 to retain some unlabeled data. Other approaches utilized the self-generated probability predictions to reweight the out-of-distribution unlabeled data. For instance, CCSSL (Wu et al., 2022) utilized probability outputs to reweight unlabeled samples. The higher the probability, the more reliable the data, and vice versa.

For consistency learning-based methods, the self-EMA (Tarvainen, 2017; Tang et al., 2021; Zhou et al., 2021) framework was designed to produce pseudo-labels under the assumption that a weighted average model can yield the reliable predictions. Some other semi-supervised learning methods allow one subnetwork to learn from another if the latter exhibits higher prediction confidence. This confidence is typically estimated based on the model's predictions (Wu et al., 2022; Hang et al., 2025), with higher confidence values indicating more reliable pseudo-labels. In this manner, the subnetworks can perform selective cross-supervision and mutually refine each other. Despite these advancements, most existing methods access the reliability of unlabeled data based on either predefined confidence thresholds or the model's prediction confidence, which limits their effectiveness in identifying truly reliable samples and makes them prone to confirmation bias.

To address above challenges, we propose WSSL, a weight-aware semi-supervised learning framework for interior decoration style classification that selectively utilizes the reliable unlabeled data. Specifically, WSSL introduces a weighting module that employs a truncated Gaussian function to automatically assess the reliability of unlabeled data. This weight module is capable of assigning higher weights to more reliable unlabeled samples during the training process. By incorporating these weights, WSSL introduces a weighted consistency (WCS) regularization that enforces prediction consistency for reliable unlabeled data under different perturbations. Besides, WSSL further introduces a weighted relation consistency (WRCS) regularization to preserve the semantic relationship consistency of reliable unlabeled samples under various perturbations. Additionally, WSSL introduces a weighted class-aware contrastive learning (WCCL) scheme to enhance the discriminative feature learning ability of model. Most importantly, experimental results show that the synergistic learning of WCS regularization, WRCS regularization, and WCCL regularization can significantly improve the model's generalizability.

Above all, the main contributions of this work are summarized as follows:

(1) We present WSSL, a novel semi-supervised framework to selectively leverage reliable unlabeled data through an adaptive weighting module to enhance interior decoration style classification.
(2) WSSL integrates three novel components: WCS regularization to enforce prediction consistency for reliable unlabeled data under different perturbations, WRCS regularization to encourage the consistency of semantic relationships among these data, and WCCL regularization to enhance the discriminative capability of the model through the use of reliable unlabeled data.
(3) The synergistic learning of WCS, WRCS, and WCCL effectively mitigates confirmation bias caused by unreliable pseudo-labels. Experimental results on interior decoration style image datasets confirm the superior performance of WSSL.

## 2 Related works

### 2.1 Deep semi-supervised learning

Semi-supervised learning methods effectively utilize the combination of limited labeled data and abundant unlabeled data to improve model performance. At present, consistency regularization and pseudo-labeling are two dominant paradigms for exploiting unlabeled data. As previously discussed, consistency learning-based methods (Tarvainen, 2017; Liu et al., 2022; Hang et al., 2025; Liu et al., 2020; Yu et al., 2019; Chen et al., 2021; Ouali et al., 2020; Miyato et al., 2018) were designed to guide the model toward producing consistent predictions for the same input under slight perturbations. These perturbations can be applied at different levels, including the input (Tarvainen, 2017), feature representations (Miyato et al., 2018), network parameters (Hang et al., 2025) or combinations of these (Liu et al., 2022). The Mean Teacher (MT) model (Tarvainen, 2017) enforces consistency by using two models with identical architectures. It employs the predictions of one model under stochastic perturbations as training targets for the other model. The parameters of the teacher network are updated through an exponential moving average (EMA) of the student network's parameters. In Hang et al. (2025), a pseudo-label-based selective mutual learning framework introduces network perturbations by utilizing two subnetworks with different architectures. Additionally, Liu et al. (2022) explores a combination of diverse perturbations to boost the generalization ability of consistency learning. Nevertheless, inaccurate predictions on unlabeled data remain a major challenge for semi-supervised learning methods, as potential mispredictions can negatively impact the consistency learning performance.

Self-training (Grandvalet and Bengio, 2004) is one of the most widely adopted strategies in pseudo-labeling-based semi-supervised learning methods, wherein pseudo-labels are assigned to unlabeled data and subsequently used together with labeled data to retrain the model. To mitigate confirmation bias induced by unreliable pseudo-labels, most semi-supervised learning methods apply a high-confidence threshold to filter out pseudo-labels with low confidence, thereby avoiding the use of potentially erroneous labels. For instance, many semi-supervised learning methods (Berthelot et al., 2019; Sohn et al., 2020; Li et al., 2020, 2023; Lee, 2013) typically use a threshold of 0.95 retain only high-confidence pseudo-labels. However, defining an optimal confidence threshold that can filter out all unreliable pseudo-labels while preserving all reliable ones remains highly challenging. A high threshold may eliminate some reliable pseudo-labels, while a low threshold increases the risk of incorporating noisy labels, potentially limiting the generalization capacity of the model.

Recently, several semi-supervised learning methods (Berthelot et al., 2019; Yu et al., 2019; Li et al., 2023; Su et al., 2024; Wu et al., 2021; Wang et al., 2023) aim to effectively utilize unlabeled data through integrating consistency learning and pseudo-labeling strategies to improve model performance. For instance, in Li et al. (2023), a diverse co-training method was devised to choose partially reliable pseudo-labels for cross-supervision between different models. Besides, Su et al. (2024) developed a mutual learning framework that utilized two subnetworks, in which the pseudo-labels with higher prediction confidence were used to guide the training of the other subnetwork. Although effective, these methods also face challenges from confirmation bias induced by potentially incorrect pseudo-labels, which can adversely impact model performance.

### 2.2 Reliable unlabeled data learning

The main challenge of semi-supervised learning methods lies in effectively identifying reliable unlabeled data, as unreliable unlabeled data adversely affect model performance. In practice, unreliable unlabeled data typically originate from two main sources: (1) out-of-distribution unlabeled data; and (2) unlabeled data associated with incorrect self-generated pseudo-labels. Current semi-supervised learning methods suffer from these unreliable unlabeled data, leading to worse performance than supervised baselines (Su et al., 2021). As models inherently produce inaccurate pseudo-labels for OOD samples, several approaches have been proposed to assess pseudo-label reliability for selecting trustworthy unlabeled data. For instance, Yao et al. (2022) proposed a confidence-aware cross pseudo supervision network that measures the confidence of pseudo-labels using KL-divergence, and then directs model focus on learning from more confident pseudo-labels. In Guo et al. (2020), pseudo-labeled unlabeled data were combined with labeled data to train the model, while performance of the supervised model was continuously monitored to prevent performance degradation. Furthermore, Hang et al. (2022) extended this strategy to construct a reliability-aware self-ensembling framework for semi-supervised classification. Following this line, this paper further explores the selective utilization of reliable unlabeled data, aiming to bridge the gap between research and practical deployment, particularly in the context of interior decoration style classification.

## 3 Weighted-aware semi-supervised self-ensembling framework

In this section, we first introduce our weight-aware module based on a truncated Gaussian function. Subsequently, we describe the proposed weighted consistency (WCS) regularization, weighted relation consistency (WRCS) regularization, and weighted class-aware contrastive learning (WCCL) regularization, respectively.

### 3.1 Problem statement

For the interior decoration style image dataset, suppose we have a small labeled training set $D^{l}={\left\{\left({\text{x}}_{i},{\text{y}}_{i}\right)\right\}}_{i=1}^{N}$, where ${\text{x}}_{i}\in X$ represents the *i*-th training image and ${\text{y}}_{i}\in Y\text{=}{\left\{\text{0,1}\right\}}^{C}$ is its corresponding ground-truth label. The total number of classes is denoted as *C*. We have an unlabeled training set $D^{u}={\left\{{\text{x}}_{i}\right\}}_{i=N+1}^{N+M}$, with *N*≪*M*. The goal is to develop a semi-supervised learning model $f:X\to {\left[0,1\right]}^{C}$ that can perform well on the test interior decoration style images.

**Figure 1:**
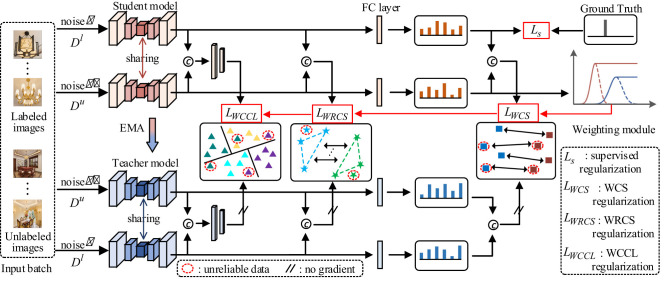
Diagram of a machine learning framework with student and teacher models, showing the flow of labeled and unlabeled images through added noise. It illustrates data sharing between models, application of regularization techniques, and a weighting module. Key components include feature extraction, comparison with ground truth, and error minimization. Annotations describe processes like EMA, unreliable data handling, and the role of various regularizations such as supervised, WCS, WRCS, and WCCL. Visuals include charts and network representations, explaining how input data is processed. The architecture of WSSL for interior decoration style classification. An input batch containing labeled and unlabeled data were fed into student and teacher network, respectively. The weighting module employs a truncated Gaussian function to automatically calculate the weights for unlabeled data. These weights were then used in WCS regularization *L*_*WCS*_, WRCS regularization *L*_*WRCS*_, and WCCL regularization *L*_*WCCL*_. Finally, the supervised loss *L*_*s*_ was combined with *L*_*WCS*_, *L*_*WRCS*_ and *L*_*WCCL*_ to optimize the model.

To address the confirmation bias induced by unreliable pseudo-labels, we devise a weight-aware semi-supervised self-ensembling (WSSL) framework, as illustrated in Figure 1. WSSL first introduces a weight module that employs a truncated Gaussian function to automatically assign higher weights to more reliable unlabeled samples during training. Based on these weights, WSSL introduces a WCS regularization to enforce consistent predictions for reliable unlabeled data under different perturbations. Besides, WSSL further introduces a WRCS regularization to ensure that the semantic relations among reliable unlabeled data remain consistent under different perturbation scenarios. Additionally, WSSL introduces a WCCL regularization to enhance the model's discriminative feature learning ability. The following sections provide detailed descriptions of the core components of WSSL, including adaptive weight learning, WCS regularization, WRCS regularization, and WCCL regularization.

### 3.2 Adaptive weight learning

To exploit the reliable unlabelled interior decoration style images, we first introduce an adaptive weight learning module that assigns weights to unlabeled data by estimating the quality of their corresponding pseudo-labels. Without loss of generality, we denote the network's prediction of an unlabeled image **x** as **p**(**y**|**x**), which is abbreviated as **p**. Thus, the adaptive weight function is presented as *g*(**p**):ℝ^*C*^ → ℝ, which maps model's output on unlabeled data to their corresponding weights. As mentioned above, relying on a fixed confidence threshold to effectively distinguish unreliable pseudo-labels from reliable ones is inherently limited, thereby constraining the model's generalization capability.

To address above issue, we assume the max value of network predictions max(**p**) follows a Gaussian distribution N(max(p),μ,σ) when max(**p**) < μ, and a uniform distribution when max(**p**)≥μ. Here, we consider the deviation of max(**p**) from the mean μ of Gaussian as a proxy measure of the correctness of pseudo-labels when max(**p**) < μ. Hence, the adaptive weight function *g*(**p**) can be formulated as a truncated Gaussian function within the range [0, λ_max_]:


(1) $$g\left(p\right)=\left\{ \begin{array}{l} \lambda_{\max}\cdot \exp\left(-\frac{{\left(\max\left(p\right)-\mu_{c}\left(t\right)\right)}^{2}}{2\sigma_{c}^{2}\left(t\right)}\right),\max\left(p\right)<\mu_{c}\left(t\right)\\ \lambda_{\max},\;\max\left(p\right)\geq \mu_{c}\left(t\right)\; \end{array}\right.$$

where μ_*c*_(*t*) and σ_*c*_(*t*), ∀*c* = 1, 2, ⋯ , *C* denote the mean and variance of the Gaussian function for the *c*-th class at the *t*-th iteration, respectively. Referring to Chen et al. (2023), the parameters λ_max_ is set to 1. Besides, we estimate μ_*c*_(*t*) and σ_*c*_(*t*) using the historical predictions of model stored in a queue list *M*. Therefore, we can obtain:


(2) $$\begin{array}{l} \mu_{c}\left(t\right)=\alpha \mu_{c}\left(t-1\right)+\left(1-\alpha \right){\hat{\mu }}_{c} \end{array}$$

(3) $$\begin{array}{l} \sigma_{c}^{2}\left(t\right)=\alpha \sigma_{c}^{2}\left(t-1\right)+\left(1-\alpha \right){\hat{\sigma }}_{c}^{2} \end{array}$$

Here, ${\hat{\mu }}_{c}=E_{M_{c}}\left[\max\left(\text{p}\right)\right]$ and ${\hat{\sigma }}_{c}^{2}=Var_{M_{c}}\left[\max\left(\text{p}\right)\right]$ denote the empirical mean and the variance of confidence of *c*-th images in *M*. Parameter α is used to balance the contributions of historical mean and the variance. In the training procedure, the queue list is iteratively update by using the images in the current batch to replace the oldest ones.

### 3.3 Weighted consistency (WCS) regularization

The proposed WSSL framework is built upon the self-ensembling paradigm, which usually takes consistency learning as regularization to exploit unlabeled data. The objective function is expressed as:


(4) $$\begin{array}{l} {\underset{\theta }{\min}}_{(x_{i},y_{i})\in D^{l}}L_{s}(f(x_{i};\theta ),y_{i})\;+\;\lambda_{x_{i}\in D^{u}}\;L_{u}(f(x_{i},\eta ;\theta ),\\ \;\;\;\;\;\;\;\;\;\;\;\;\;\;\;\;\;\;\;\;\;\;\;\;\;\;\;\;\;\;\;\;\;\;\;\;\;\;\;\;\;\;\;\;\;\;\;\;\;\;\;\;\;\;\;\;\;\;\;\;\;\;\;\;\;\;\;\;\;\;\;f(x_{i},\eta^{\prime };\theta^{\prime })) \end{array}$$

Here, *L*_*s*_ is the supervised loss, while*L*_*u*_ represents the unsupervised consistency loss (e.g., mean squared error), which is utilized for exploiting the unlabeled data. θ and θ′ refer to the student and teacher network parameters, respectively. Besides, η and η′ denote the different input perturbations, such as random rotations, translations, and horizontal flips. According to the self-ensembling principle (Tarvainen, 2017), the parameters of the teacher network are updated through EMA of the student network's parameters.

It can be observed from Equation 4 that all unlabeled data are treated equally during model training, without considering the potential negative impact of unreliable unlabeled data on model performance. To address this limitation, we incorporate the adaptive weight learning module to assign differentiated weights to unlabeled data. We then propose a weighted consistency (WCS) regularization *L*_*WCS*_, which selectively enforces consistency on reliable unlabeled data, which can be formulated as:


(5) $$L_{WCS}{=}_{x_{i}\in D^{u}\;\;}w_{i}\cdot L_{u}(f(x_{i},\eta ;\theta ),f(x_{i},\eta^{\prime };\theta^{\prime }))$$

where the weights *w*_*i*_ = *g*(**p**_*i*_), *i* = *N*+1, *N*+2, ⋯ , *N*+*M* can be automatically estimated from Equation 1. It can be seen from Equation 5, the proposed WCS regularization enforces consistency among ensemble predictions for reliable unlabeled samples under different input perturbations.

### 3.4 Weighted relation consistency (WRCS) regularization

The above WCS regularization only focuses on individual reliable data points, we argue that the relationships between reliable data should also remain consistent under different perturbations. In view of this, we design a weighted sample relation consistency (WRCS) regularization to model the intrinsic relationships among reliable data. The proposed WRCS encourages the network to keep consistent semantic relationships between samples under different perturbations, enabling the model extract inherent semantic information from reliable unlabeled data.

Assuming the input min-batch **x**^*B*^ contains *B* samples, the feature representations are expressed as *F*∈ℝ^*B*×*C*×*H*×*W*^. *H*, *W* and *C* denote height, width and number of channels. We reshape *F* to *E*∈ℝ^*B*×*CHW*^, and compute the sample relation matrix *G* as the Gram Matrix of *E*:


(6) $$\begin{array}{l} G=E\cdot E^{T}\in {\mathbb{R}}^{B\times B} \end{array}$$

Here, the (*i, j*)-th term *G*_*i, j*_ denotes the inner product of vectorized features *E*_*i*_ and *E*_*j*_. Equation 6 can thus be interpreted as a similarity measure between the feature maps of the *i*-th and *j*-th samples. We then use L2 normalization to each row of sample relation matrix *G*:


(7) $$\begin{array}{l} R={\left[\frac{G_{1}}{{\left||G_{1}|\right|}_{2}},\frac{G_{2}}{{\left||G_{2}|\right|}_{2}},\cdots \;,\frac{G_{B}}{{\left||G_{B}|\right|}_{2}}\right]}^{T} \end{array}$$

Finally, the WRCS regularization used to preserve the semantic relationships under different perturbations can be formulated as:


(8) $$\begin{array}{l} L_{WRCS}=\frac{1}{B}\cdot \text{W}\cdot ||R\left({\text{x}}^{B},\eta ;\theta \right)-R\left({\text{x}}^{B},\eta^{\prime };\theta^{\prime }\right)|{|}_{2}^{2} \end{array}$$

Here, **W** = [*w*_1_, *w*_2_, ⋯ , *w*_*N*+*M*_] subsumes ${\text{W}}_{l}\in {\mathbb{R}}_{=1}^{N}$ and ${\text{W}}_{u}\in {\mathbb{R}}_{\leq \lambda_{\max}}^{M}$. Besides, the weights **W**_*u*_ is automatically computed by using Equation 1. *L*_*WRCS*_ can help maintain the intrinsic relationships among samples across various perturbations, thereby facilitating the extraction of additional semantic information within unlabeled data.

### 3.5 Weighted class-aware contrastive learning (WCCL) regularization

Supervised contrastive learning (Zhang et al., 2022; Li et al., 2021) has been commonly employed in semi-supervised learning framework to capture pairwise relationships among unlabeled samples based on their pseudo-labels. However, the incorrect pseudo-labels may degrade the effectiveness of contrastive learning. To address this issue, we devise a weighted class-aware contrastive learning (WCCL) regularization, which selectively emphasizes reliable unlabeled samples to promote the discriminative feature learning capability of model.

Formally, the classical supervised contrastive learning scheme is expressed as:


(9) $$L_{CCL}{=}_{i\in Q}{\frac{1}{J+1}}_{j\in P(i)}-\log\frac{\exp(z_{i}\cdot {\hat{z}}_{j}/\tau \;)}{Pos+Neg}$$

(10) $$Pos=\exp\left(z_{i}\cdot {\hat{z}}_{i}/\tau \;\right){+}_{j\in P\left(i\right)\backslash \left\{i\right\}}\exp\left(z_{i}\cdot {\hat{z}}_{j}/\tau \;\right)$$

(11) $$Neg{=}_{j\in Q\backslash P\left(i\right)}\exp\left(z_{i}\cdot {\hat{z}}_{j}/\tau \;\right)$$

In this context, **z**_*i*_ and ${\hat{\text{z}}}_{i}$, ∀*i* = 1, 2, ⋯*M* are the normalized embeddings produced by the projection networks *h* and ĥ, denoted as **z**_*i*_ = *h*(*f*(**x**_*i*_, η; θ)) and ${\hat{\text{z}}}_{i}=ĥ\left(f\left({\text{x}}_{i},\eta^{\prime };\theta^{\prime }\right)\right)$. τ is a hyper-parameter. *Q* is a dynamic queue list. *P*(*i*) refers to the set of indices for all positive samples based on their pseudo-labels.

Although above supervised contrastive learning strategy is effective, the unreliable unlabeled data may impair the classification performance of model. Hence, we devise a weighted contrastive learning (WCCL) regularization, which can be expressed as:


(12) $$L_{WCCL}={-}_{i\in Q}\;{\frac{1}{J+1}}_{j\in P(i)}w_{i}\cdot \log\frac{\exp(z_{i}\cdot {\hat{z}}_{j}/\tau \;)}{Pos+Neg}$$

Notably, *L*_*WCCL*_ will degenerate to *L*_*CCL*_ when *w*_*i*_ of each unlabeled data tends to 1. It can be seen that *L*_*WCCL*_ selectively leverages the reliable unlabeled data, thereby enhancing the effectiveness of contrastive learning.

### 3.6 Objective function

Overall, the objective of our weight-aware semi-supervised self-ensembling (WSSL) framework can be formulated as:


(13) $$\begin{array}{l} L=L_{s}+\lambda_{1}\cdot L_{WCS}+\lambda_{2}\cdot L_{WRCS}+\lambda_{3}\cdot L_{WCCL} \end{array}$$

Here, *L*_*s*_ denotes the supervised cross-entropy loss. λ_1_, λ_2_, and λ_3_ are hyper-parameters that are used to balance the regularizations. The optimization procedure of our WSSL is given in Algorithm 1 in detail.

**Algorithm 1:**
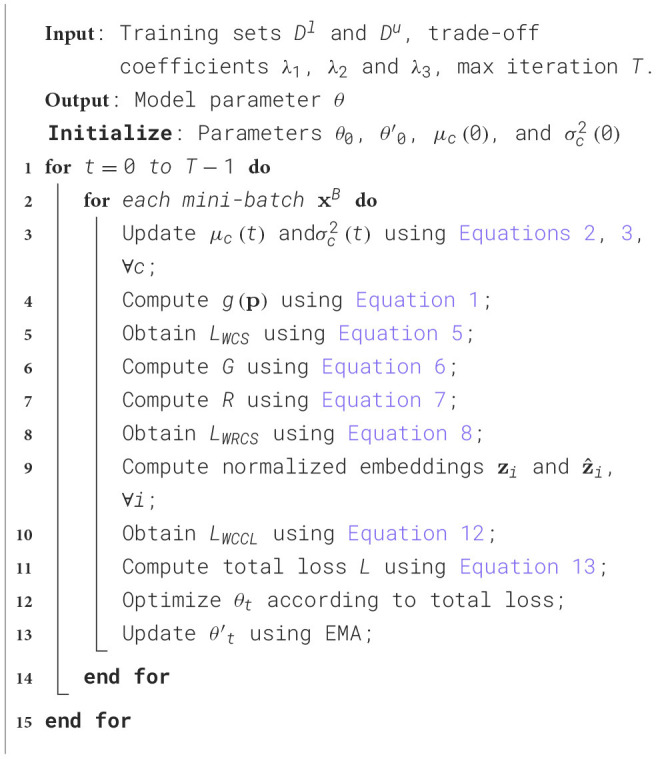
Optimization of WSSL framework.

## 4 Experiments

This section presents the evaluation of WSSL on five distinct interior decoration style image datasets, including: (1) TV background wall, (2) chandelier, (3) living room, (4) dining room, and (5) bedroom. Initially, we provided a detailed description of these datasets. Then, we introduce the comparative semi-supervised learning methods and outline the corresponding implementation details. Finally, we report and analyze the experimental results.

### 4.1 Datasets

#### 4.1.1 TV background wall

The dataset comprises 1,643 images, categorized into four types: 250 for Country style, 270 for Chinese style, 322 for European style, and 801 for Simple style.

#### 4.1.2 Chandelier

The dataset includes 969 images, distributed across four style categories: 295 for Country style, 169 for Chinese style, 351 for European style, and 154 for Simple style.

#### 4.1.3 Living room

The dataset comprises 1,489 images: 138 for Country style, 248 for Chinese style, 523 for European style, and 580 for Simple style.

#### 4.1.4 Dining room

The dataset contains 520 images: 91 for Country style, 98 for Chinese style, 178 for European style, and 153 for Simple style.

#### 4.1.5 Bedroom

The dataset includes 643 images: 149 for Country style, 119 for Chinese style, 191 for European style, and 184 for Simple style.

All images were resized to a resolution of 900 × 700 pixels. Besides, each of the five datasets used in this study was individually partitioned at random into training (70%), validation (10%), and testing (20%) sets.

### 4.2 Experimental setup

To assess the performance of WSSL for interior decoration style classification, we compared WSSL with several semi-supervised learning methods. The comparison methods include: (1) DenseNet121 (Huang et al., 2017); (2) MT model (Tarvainen, 2017); (3) MixMatch (Berthelot et al., 2019); (4) UDA (Xie et al., 2020); (5) FlexMatch (Zhang et al., 2021); (6) SimMatch (Zheng et al., 2022); (7) SRC-MT (Liu et al., 2020); (8) the proposed weight-aware semi-supervised learning framework (WSSL).

In the experiment, DenseNet121 was employed as the backbone for all the comparison methods. For pseudo-labeling-based semi-supervised learning methods MixMatch, FlexMatch and SimMatch, we refer to the original work to apply the weak and strong augmentations on the input images. Besides, the confidence value was set to 0.95. In consistency learning-based semi-supervised learning methods MT, SRC-MT and our WSSL, a series of random transformations were used as the perturbations for the input images, including rotation, translation, and horizontal/vertical flips. Specifically, the random rotation angle was configured to span from −10 degrees to 10 degrees Hang et al., (2024). For image translation, the number of pixels for both horizontal and vertical displacements was defined within ±2% of the image width. Moreover, input images were subjected to random horizontal and vertical flips with a probability of 50%. Besides, parameter of EMA was set to 0.99. For our WSSL, the hyper-parameters λ_1_, λ_2_, and λ_3_ assigned values of 0.7, 0.7 and 0.1, respectively. Model training was conducted using the Adam optimizer for 10K iterations, with a fixed learning rate of 0.0001. Each training batch comprised 24 samples, equally divided into 12 labeled and 12 unlabeled images. Other experimental settings for the comparison methods followed their original implementations. Our WSSL was implemented using two RTX 3090 GPUs.

In the experiments, AUC, accuracy (ACC), sensitivity (SEN), precision (PREC), and F1 score (F1) were utilized as the evaluation metrics to assess the classification performance of compared methods.

### 4.3 Comparison with state-of-the-art methods

Tables 1–5 present the classification results of comparison methods trained with 20% labeled and 80% unlabeled training images across five interior decoration style image datasets. The results obtained by DenseNet121 trained with 100% labeled data are considered as the upper-bound performance, while the performance of DenseNet121 trained solely on 20% labeled data is regarded as the baseline. Among all classification results, we highlighted the best results in bold, as shown in Tables 1–5. For clarity, the performance improvements relative to the baseline are indicated in parentheses. According to the classification results presented in Tables 1–5, we can make the following observations.

**Table 1:** Performance (%) comparison with different semi-supervised learning methods on TV background wall dataset.

**Methods**	**Percentage**		**Metrics**				
	**Labeled**	**Unlabeled**	**ACC**	**AUC**	**SEN**	**PREC**	**F1**
DenseNet121; Huang et al. (2017)	100%	0	94.04	97.44	86.12	86.18	86.08
DenseNet121; Huang et al. (2017)	20%	0	88.53	93.76	73.84	72.73	72.90
MT; Tarvainen (2017)	20%	80%	90.06 (1.53)	95.55 (1.79)	74.03 (0.19)	81.07 (8.34)	75.98 (3.08)
MixMatch; Berthelot et al. (2019)	20%	80%	90.83 (2.30)	94.77 (1.01)	78.98 (5.14)	80.85 (8.12)	79.48 (6.58)
UDA; Xie et al. (2020)	20%	80%	90.78 (2.25)	94.90 (1.14)	79.05 (5.21)	81.10 (8.37)	79.46 (6.56)
FlexMatch; Zhang et al. (2021)	20%	80%	91.30 (2.77)	95.59 (1.83)	79.97 (6.13)	80.96 (8.23)	79.13 (6.23)
SimMatch; Zheng et al. (2022)	20%	80%	91.92 (3.39)	95.21 (1.45)	79.40 (5.56)	81.18 (8.45)	79.44 (6.54)
SRC-MT; Liu et al. (2020)	20%	80%	91.36 (2.83)	95.84 (2.08)	80.11 (6.27)	81.33 (8.60)	79.76 (6.86)
WSSL (Ours)	20%	80%	93.26 (4.73)	96.73 (2.97)	81.00 (7.16)	84.01 (11.2)	80.51 (7.61)

**Table 2:** Performance (%) comparison with different semi-supervised learning methods on chandelier dataset.

**Methods**	**Percentage**		**Metrics**				
	**Labeled**	**Unlabeled**	**ACC**	**AUC**	**SEN**	**PREC**	**F1**
DenseNet121; Huang et al. (2017)	100%	0	97.28	99.61	95.58	93.85	94.61
DenseNet121; Huang et al. (2017)	20%	0	87.69	93.96	78.75	77.72	76.53
MT; Tarvainen (2017)	20%	80%	92.75 (5.06)	96.38 (2.42)	87.45 (8.70)	83.50 (5.78)	83.70 (7.17)
MixMatch; Berthelot et al. (2019)	20%	80%	91.58 (3.89)	95.75 (1.79)	84.72 (5.97)	83.14 (5.42)	83.63 (7.10)
UDA; Xie et al. (2020)	20%	80%	91.68 (3.99)	96.08 (2.12)	87.99 (9.24)	84.03 (6.31)	85.50 (8.97)
FlexMatch; Zhang et al. (2021)	20%	80%	92.09 (4.40)	96.75 (2.79)	88.27 (9.52)	84.55 (6.83)	84.32 (7.79)
SimMatch; Zheng et al. (2022)	20%	80%	92.28 (4.59)	97.39 (3.43)	88.64 (9.89)	84.98 (7.26)	84.54 (8.01)
SRC-MT; Liu et al. (2020)	20%	80%	92.49 (4.80)	97.35 (3.39)	88.66 (9.91)	86.38 (8.66)	84.07 (7.54)
WSSL (Ours)	20%	80%	94.30 (6.61)	98.76 (4.80)	98.30 (19.5)	87.26 (9.54)	89.16 (12.6)

**Table 3:** Performance (%) comparison with different semi-supervised learning methods on living room dataset.

**Methods**	**Percentage**		**Metrics**				
	**Labeled**	**Unlabeled**	**ACC**	**AUC**	**SEN**	**PREC**	**F1**
DenseNet121; Huang et al. (2017)	100%	0	92.42	94.31	83.30	83.91	83.52
DenseNet121; Huang et al. (2017)	20%	0	86.95	89.74	69.34	69.45	67.28
MT; Tarvainen (2017)	20%	80%	87.96 (1.01)	91.91 (2.17)	71.77 (2.43)	73.74 (4.29)	69.7 (2.42)
MixMatch; Berthelot et al. (2019)	20%	80%	88.89 (1.94)	91.26 (1.52)	72.91 (3.57)	77.07 (7.62)	73.36 (6.08)
UDA; Xie et al. (2020)	20%	80%	88.53 (1.58)	90.82 (1.08)	71.35 (2.01)	73.87 (4.42)	69.78 (2.50)
FlexMatch; Zhang et al. (2021)	20%	80%	88.43 (1.48)	91.88 (2.14)	71.82 (2.48)	74.54 (5.09)	68.10 (0.82)
SimMatch; Zheng et al. (2022)	20%	80%	88.22 (1.27)	92.63 (2.89)	71.62 (2.28)	74.01 (4.56)	69.10 (1.82)
SRC-MT; Liu et al. (2020)	20%	80%	88.22 (1.27)	92.41 (2.67)	71.98 (2.64)	74.23 (4.78)	70.43 (3.15)
WSSL (Ours)	20%	80%	90.38 (3.43)	92.85 (3.11)	85.87 (16.5)	77.15 (7.70)	76.34 (9.06)

**Table 4:** Performance (%) comparison with different semi-supervised learning methods on dining room dataset.

**Methods**	**Percentage**		**Metrics**				
	**Labeled**	**Unlabeled**	**ACC**	**AUC**	**SEN**	**PREC**	**F1**
DenseNet121; Huang et al. (2017)	100%	0	94.71	98.03	91.66	89.27	90.2
DenseNet121; Huang et al. (2017)	20%	0	87.26	91.89	76.37	74.64	75.24
MT; Tarvainen (2017)	20%	80%	88.46 (1.20)	93.97 (2.08)	81.76 (5.39)	76.00 (1.36)	77.65 (2.41)
MixMatch; Berthelot et al. (2019)	20%	80%	89.18 (1.92)	95.20 (3.31)	79.68 (3.31)	80.20 (5.56)	78.60 (3.36)
UDA; Xie et al. (2020)	20%	80%	90.72 (3.46)	94.30 (2.41)	81.67 (5.30)	76.58 (1.94)	78.18 (2.94)
FlexMatch; Zhang et al. (2021)	20%	80%	91.55 (4.29)	93.86 (1.97)	82.07 (5.70)	77.49 (2.85)	78.09 (2.85)
SimMatch; Zheng et al. (2022)	20%	80%	92.00 (4.74)	94.18 (2.29)	83.11 (6.74)	76.79 (2.15)	78.44 (3.20)
SRC-MT; Liu et al. (2020)	20%	80%	91.11 (3.85)	95.45 (3.56)	85.17 (8.80)	77.86 (3.22)	79.53 (4.29)
WSSL (Ours)	20%	80%	94.47 (7.21)	98.97 (7.08)	95.10 (18.7)	85.81 (11.1)	88.00 (12.8)

**Table 5:** Performance (%) comparison with different semi-supervised learning methods on bedroom dataset.

**Methods**	**Percentage**		**Metrics**				
	**Labeled**	**Unlabeled**	**ACC**	**AUC**	**SEN**	**PREC**	**F1**
DenseNet121; Huang et al. (2017)	100%	0	85.74	88.40	72.55	72.35	71.95
DenseNet121; Huang et al. (2017)	20%	0	78.71	81.35	57.78	59.35	56.82
MT; Tarvainen (2017)	20%	80%	80.86 (2.15)	84.44 (3.09)	64.05 (6.27)	61.37 (2.02)	61.67 (4.85)
MixMatch; Berthelot et al. (2019)	20%	80%	81.84 (3.13)	84.04 (2.69)	65.42 (7.64)	63.59 (4.24)	64.01 (7.19)
UDA; Xie et al. (2020)	20%	80%	80.05 (1.34)	84.23 (2.88)	62.57 (4.79)	61.18 (1.83)	61.35 (4.53)
FlexMatch; Zhang et al. (2021)	20%	80%	79.97 (1.26)	85.09 (3.74)	61.97 (4.19)	60.08 (0.73)	62.12 (5.30)
SimMatch; Zheng et al. (2022)	20%	80%	80.33 (1.62)	85.76 (4.41)	62.47 (4.69)	61.16 (1.81)	61.00 (4.18)
SRC-MT; Liu et al. (2020)	20%	80%	80.08 (1.37)	86.01 (4.66)	60.23 (2.45)	61.64 (2.29)	60.04 (3.22)
WSSL (Ours)	20%	80%	82.03 (3.32)	91.20 (9.85)	74.25 (16.4)	64.67 (5.32)	69.61 (12.8)

Overall, the semi-supervised learning methods consistently outperform baseline model by leveraging both labeled and unlabeled data during training. Besides, almost all semi-supervised learning methods fail to surpass the upper-bound classification results obtained by DenseNet 121 trained on 100% labeled data. This performance gap is primarily attributed to the adverse impact of inaccurate self-generated pseudo-labels on model training. WSSL exhibits superior classification performance compared to other semi-supervised methods, confirming the efficacy of our weight-aware semi-supervised learning framework. Most importantly, WSSL even exceeds the upper-bound performance on certain evaluation metrics, demonstrating its effectiveness in selectively exploiting reliable unlabeled data through the proposed adaptive weighting module. For example, our WSSL consistently achieves higher SEN and AUC scores than DenseNet121 trained on 100% labeled data across the majority of datasets. Besides, WSSL closely approaches the upper-bound classification results across multiple evaluation metrics. These results further underscore the benefits of selectively leveraging reliable unlabeled data, which also explains the performance gains achieved by our method.

**Figure 2:**
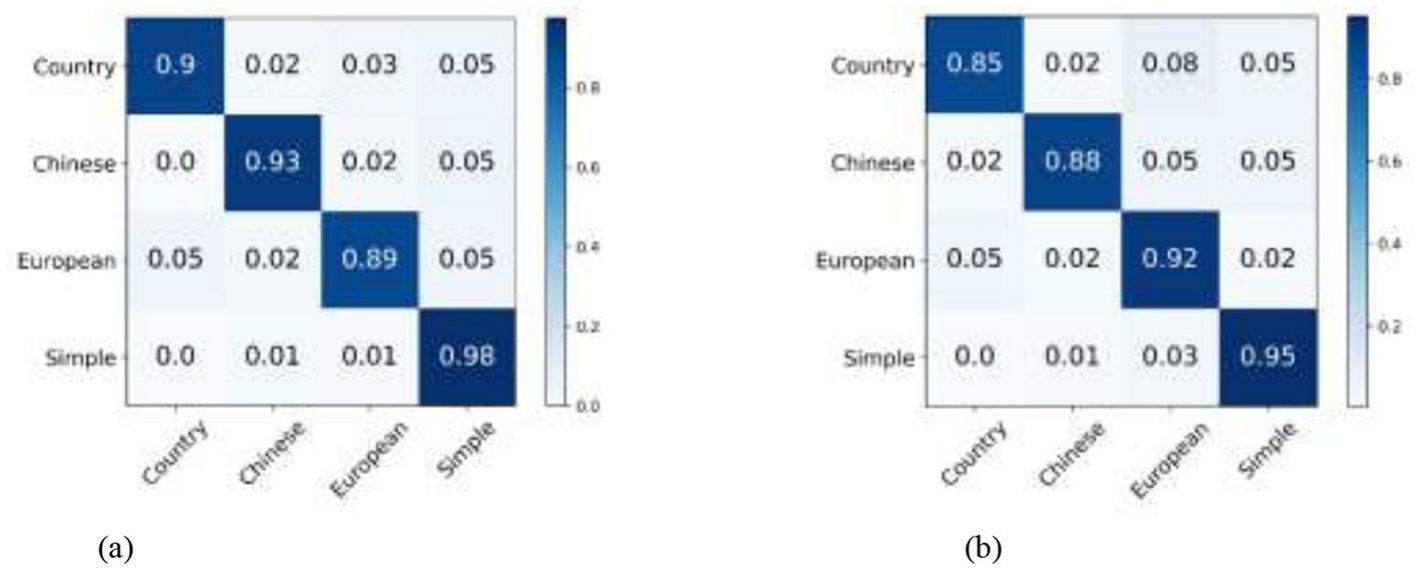
Two confusion matrices compare datasets. (a) TV Background Wall dataset shows high accuracy with values on the diagonal: Country 0.9, Chinese 0.93, European 0.89, Simple 0.98. (b) Living Room dataset shows similar accuracy: Country 0.85, Chinese 0.88, European 0.92, Simple 0.95. Non-diagonal values are lower, indicating classification errors. Color intensity represents value magnitude. Confusion matrix of WSSL for interior decoration style classification: **(a)** TV background wall dataset, and **(b)** Living room dataset.

Specifically, our WSSL achieves notable performance gains over the baseline across five image datasets, with average improvements of 5.06%, 5.56%, 15.65%, 8.97%, and 10.97% in terms of ACC, AUC, SEN, PREC, and F1, respectively. When compared to the most competitive consistency learning-based semi-supervised learning method SRC-MT, WSSL obtained the classification improvements of 2.24%, 2.29%, 9.64%, 3.46%, and 5.96%, respectively. Similarly, compared to the most competitive pseudo-labeling-based semi-supervised learning method SimMatch, WSSL demonstrates respective gains of 1.94%, 2.67%, 9.82%, 4.13%, and 6.22%, respectively. The encouraging classification results highlight the efficacy of the synergistic integration of the proposed weighted consistency regularization, weighted relation consistency regularization, and weighted class-aware contrastive learning regularization. In addition, for each interior decoration style image dataset, our WSSL consistently outperforms other comparative methods on all evaluation metrics. For instance, WSSL outperforms the strongest competing method, SRC-MT, on the TV background wall dataset, with improvements of 1.9%, 0.89%, 0.89%, 2.6%, and 0.75% in terms of ACC, AUC, SEN, PREC, and F1, respectively. On the Bedroom dataset, WSSL outperformed the most competitive method, MixMatch, with improvements of 0.19%, 7.16%, 8.76%, 1.08%, and 5.61% across various evaluation metrics. To analyze the classification performance across individual categories, we present the confusion matrices of the proposed WSSL on two class-imbalanced datasets, i.e., TV Background Wall dataset and Living Room dataset, as shown in Figure 2. It can be observed that the diagonal elements in both confusion matrices remain relatively balanced, indicating that WSSL achieves stable performance across different classes. Although WSSL primarily focuses on selecting reliable unlabeled samples rather than explicitly handling class imbalance, it still demonstrates promising classification performance on these imbalanced datasets. These enhancements further demonstrate the benefits of incorporating the weight-aware strategy into semi-supervised learning framework.

To further evaluate the statistical significance of classification results, we conducted pairwise two-tailed *t*-tests to determine whether the performance of WSSL is significantly superior to that of other semi-supervised methods in interior decoration style image classification. The statistical outcomes across five datasets are summarized in Table 6, where *p*-values less than 0.05 are highlighted. As observed, in most cases, the null hypothesis can be rejected at a 95% confidence level, which means the observed performance gains of WSSL over comparison methods are statistically significant.

**Table 6:** Statistical significance comparisons of different metrics of WSSL and other methods on five datasets.

**Methods**	***p*** **-values**				
	**ACC**	**AUC**	**SEN**	**PREC**	**F1**
WSSL vs. MT	**2.89E-02**	**4.57E-02**	**9.29E-04**	**2.32E-02**	**2.36E-03**
WSSL vs. MixMatch	**4.50E-02**	**2.44E-02**	**1.15E-02**	**4.87E-02**	**2.54E-02**
WSSL vs. UDA	**1.66E-03**	**2.02E-02**	**9.59E-03**	**2.13E-02**	**2.05E-02**
WSSL vs. FlexMatch	**2.55E-04**	**4.49E-02**	**1.29E-02**	**1.68E-02**	**1.29E-02**
WSSL vs. SimMatch	**5.65E-04**	6.05E-02	**1.08E-02**	**2.80E-02**	**1.54E-02**
WSSL vs. SRC-MT	**1.46E-03**	6.30E-02	**1.54E-02**	**4.17E-02**	**1.79E-02**

Bold indicates the *p*-values which are less than 0.05.

### 4.4 Evaluation of the proposed WSSL framework

#### 4.4.1 Efficacy of different components

To further access the classification efficacy of the proposed WSSL, we conducted the ablation study for evaluating the individual contributions of its components. Without loss of generality, the classification performance obtained using different components on the Chandelier and Dining Room datasets were presented in Tables 7, 8. In the experiment, we utilized the following models: (a) DenseNet121 (Scenario 1); (b) MT framework with DenseNet121 as the backbone network (Scenario 2); (c) MT framework with WCS regularization (Scenario 3); (d) MT framework with WCS regularization and WRCS regularization (Scenario 4); (e) MT framework with WCS regularization and WCCL regularization (Scenario 5); (f) MT framework with WCS regularization, WRCS regularization, and WCCL regularization (Scenario 6, WSSL).

**Table 7:** Ablation study of different components on the classification performance of chandelier dataset.

**DenseNet 121**	**MT**	**WCS**	**WRCS**	**WCCL**	**Chandelier dataset**				
					**ACC**	**AUC**	**SEN**	**PREC**	**F1**
√					87.69	93.96	78.75	77.72	76.53
√	√				92.75	96.38	87.45	83.50	86.70
√	√	√			93.26	97.12	90.85	84.30	87.01
√	√	√	√		93.90	97.74	95.37	84.21	87.67
√	√	√		√	93.13	97.31	94.74	84.36	87.43
√	√	√	√	√	94.30	98.76	98.30	87.26	89.16

**Table 8:** Ablation study of different components on the classification performance of dining room dataset.

**DenseNet 121**	**MT**	**WCS**	**WRCS**	**WCCL**	**Dining room dataset**				
					**ACC**	**AUC**	**SEN**	**PREC**	**F1**
√					87.26	91.89	76.37	74.64	75.24
√	√				88.46	93.97	81.76	76.00	77.65
√	√	√			89.98	95.64	86.24	76.11	78.32
√	√	√	√		90.38	95.77	86.75	77.11	81.15
√	√	√		√	90.38	96.43	87.43	76.61	80.71
√	√	√	√	√	94.47	98.97	95.10	85.81	88.00

As demonstrated in Tables 7, 8, the classification performance in Scenario 2 outperforms Scenario 1, confirming MT can effectively leverage unlabeled data. The classification results of Scenario 3 surpass those of Scenario 2, providing empirical evidence for the efficacy of the proposed WCS. Besides, the classification results of Scenario 4 and Scenario 5 surpass Scenario 3, further verifying the effectiveness of the proposed WRCS and WCCL regularizations. We then integrated WCS, WRCS, and WCCL regularizations into the MT framework, *i.e*., Scenario 6, leading to further enhancements of classification performance. These results confirm the effectiveness of weight-aware mechanism to use the reliable unlabeled data.

**Figure 3:**
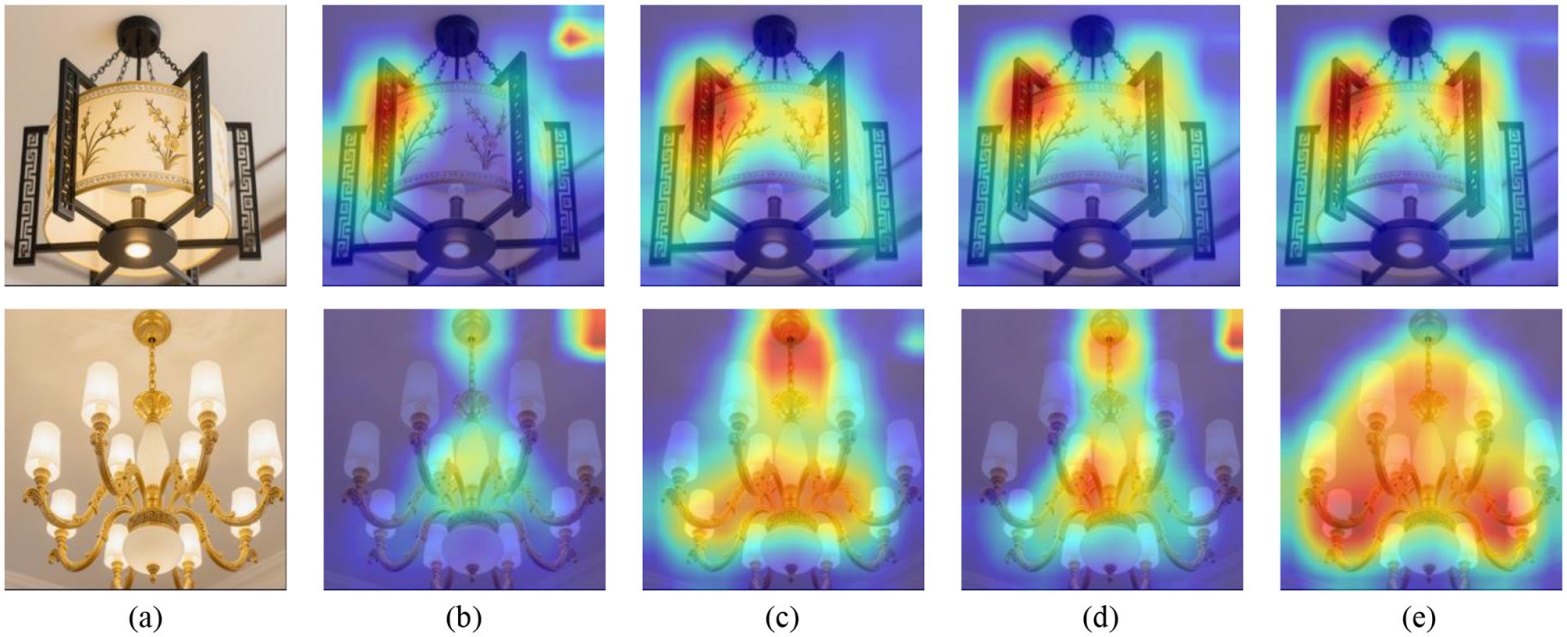
Top row shows five images of a hanging lantern, with the first being a clear photo and the next four displaying heat maps indicating areas of interest. Bottom row features similar sequence with chandeliers, beginning with a clear image followed by four heat maps highlighting different focal points. Grad-CAM visualizations of attention regions for interior decoration style image from the Chandelier dataset. **(a)** Original images, **(b)** Scenario 3, **(c)** Scenario 4, **(d)** Scenario 5, and **(e)** Scenario 6. The first and the second row denote Chinese style and European style, respectively.

**Figure 4:**
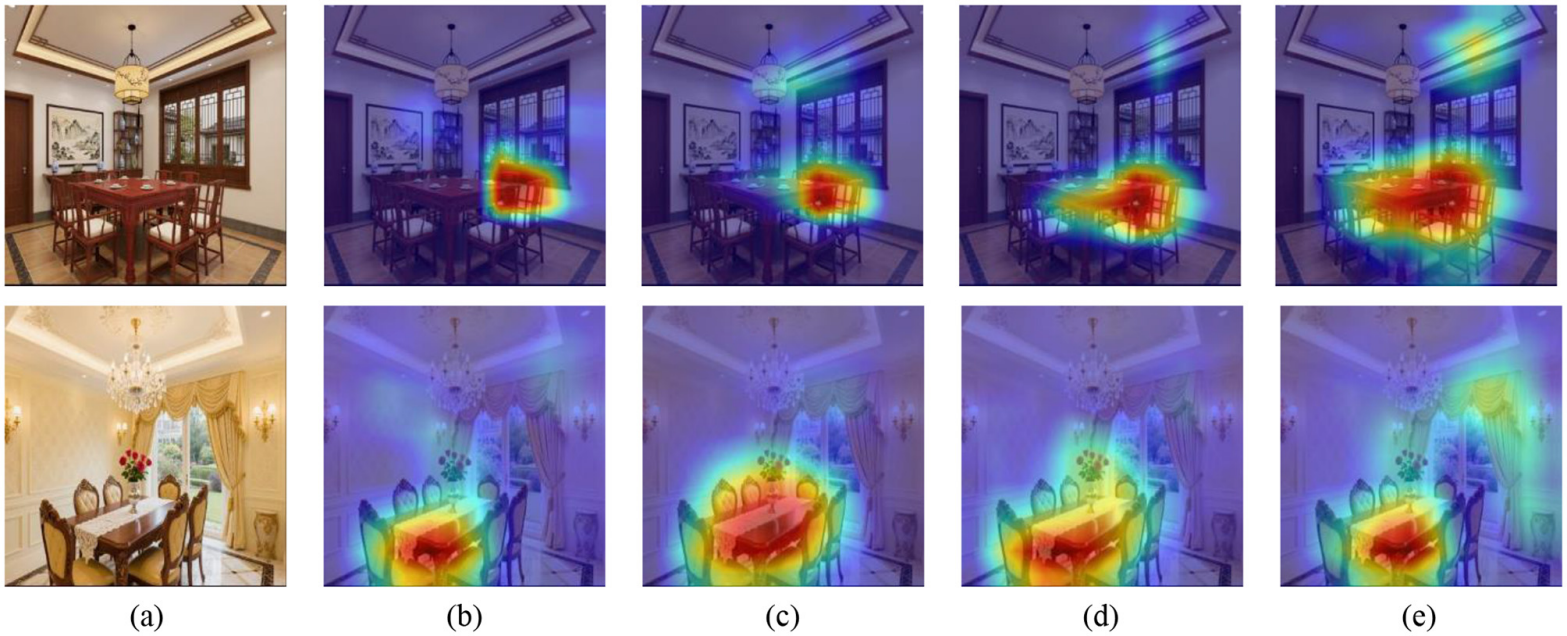
Two rows of images compare original photographs and heatmaps. The top row shows an interior dining room with the original image (a) followed by heatmaps (b-e) indicating areas of interest in varying colors from blue to red. The bottom row features another dining room with the original image (a), followed by similar heatmaps (b-e), highlighting different focal points. Grad-CAM visualizations of attention regions for interior decoration style image from the dining room dataset. **(a)** Original images, **(b)** Scenario 3, **(c)** Scenario 4, **(d)** Scenario 5, and **(e)** Scenario 6. The first and the second row denote Chinese style and European style, respectively.

Furthermore, we adopted Gradient Weighted Class Activation Mapping (Grad-CAM) Selvaraju et al., (2020) to visualize the salient regions in the image that significantly impact the model's prediction for specific classes. To further examine the effectiveness of different components, we presented the Grad-CAMs of Scenarios 3–6, as illustrated in Figures 3, 4. As observed, the first and second row displays images in Chinese and the European styles, respectively. Notably, the chandelier serves as a representative style attribute that reflects the overall interior decoration category. The salient regions in Figures 3b, 4b indicate only partial stylistic cues (i.e., chandelier) identified under the MT framework with WCS regularization. Moreover, the highlighted regions in Figures 3c, 4c and Figures 3d, 4d are more accurately localized than those in Figures 3b, 4b, confirming the effectiveness of WRCS regularization and WCCL regularization, respectively. Notably, Figures 3e, 4e demonstrate that WSSL can accurately localize multiple chandeliers that correspond to distinct style attributes. These visualizations suggest that WSSL not only captures stylistic variation more effectively but also emphasizes the discriminative characteristics inherent to each style category. For a human design perspective, designers often use lighting elements (*e.g*., chandelier) to define and differentiate interior styles, indicating that the model's attention aligns well with human design logic. Overall, this attention maps from Grad-CAM visualizations not only demonstrate the model's ability to capture discriminative, style-specific features but also provide valuable interpretability and guidance for downstream applications in real-world interior design.

#### 4.4.2 Impact of different percentage of labeled data

To investigate the classification performance of WSSL under varying proportions of annotated images, we conducted experiments using 5%, 10%, and 20% of annotated training images. We compared WSSL with three representative approaches, *i.e*., the baseline method DenseNet 121, the most competitive pseudo-labeling-based method SimMatch, and the most competitive consistency learning-based method RAC-MT. The classification results using different percentage of annotated images were listed in Table 9. Overall, the classification performance of all methods continues to improve as the proportion of annotated images increases. Moreover, the classification performance of the proposed WSSL consistently outperforms other comparison methods across different proportion of annotated images, further validating the scalability of weight-aware self-ensembling framework. The results verify the efficacy of the weighting mechanism and highlight the critical role of using reliable unlabeled images in both semantic learning and semantic relationship modeling.

**Table 9:** Performance (%) of comparison methods using different percentage of labeled data on two datasets.

**Percentage**	**Methods**	**Chandelier dataset**					**Dining room dataset**				
		**ACC**	**AUC**	**SEN**	**PREC**	**F1**	**ACC**	**AUC**	**SEN**	**PREC**	**F1**
5%	DenseNet 121	75.16	88.56	76.74	57.17	70.68	78.61	87.85	72.20	55.39	61.19
	SimMatch	78.39	90.30	85.77	62.66	72.31	82.48	91.49	80.39	59.16	62.80
	RAC-MT	85.52	91.06	82.53	60.59	74.79	82.28	92.10	82.27	60.84	63.27
	WSSL	**88.02**	**92.80**	**92.10**	**65.19**	**78.75**	**85.52**	**93.49**	**92.38**	**62.67**	**65.58**
10%	DenseNet 121	82.97	90.53	77.75	68.34	72.81	81.25	88.22	74.02	59.85	66.47
	SimMatch	87.99	95.02	87.22	70.02	78.58	85.58	93.77	82.56	66.49	73.20
	RAC-MT	87.50	96.29	86.72	71.30	80.84	86.53	94.17	84.04	65.69	72.56
	WSSL	**89.77**	**97.46**	**95.22**	**73.57**	**82.65**	**87.74**	**96.79**	**93.08**	**67.14**	**78.66**
20%	DenseNet 121	87.69	93.96	78.75	77.72	76.53	87.26	91.89	76.37	74.64	75.24
	SimMatch	92.28	97.39	88.64	84.98	84.54	92.00	94.18	83.11	76.79	78.44
	RAC-MT	92.49	97.35	88.66	86.38	84.07	91.11	95.45	85.17	77.86	79.53
	WSSL	**94.30**	**98.76**	**98.30**	**87.26**	**89.16**	**94.47**	**98.97**	**95.10**	**85.81**	**88.00**

Highest classification accuracy was highlighted in bold.

#### 4.4.3 Impact of hyper-parameters

The impact of hyper-parameters on the classification performance of WSSL were also investigated. WSSL has three hyper-parameters, *i.e*., λ_1_, λ_2_, andλ_3_, where λ_1_ is the weight for WCS regularization, λ_2_ is the weight for WRCS regularization, and λ_3_ is the weight for WCCL regularization. The two parameters were held constant while the third was adjusted to evaluate its impact on the classification performance of WSSL, as shown in Table 10. When λ_1_ = λ_2_ = 0.7, the classification performance improves as the hyper-parameter λ_3_ increases, indicating that assigning greater emphasis to the WCCL regularization can effectively enhance the classification results. However, as λ_3_ continues to increase, the classification performance degrades, indicating that neglecting the other two regularizations may negatively impact classification performance. For hyper-parameters λ_2_ andλ_3_, the similar results can be found, as shown in Table 10. These results demonstrate the robustness of the proposed weight-aware semi-supervised self-ensembling framework.

**Table 10:** Sensitivity of trade-off coefficients on the classification performance (%) on two datasets.

**λ_1_ = 0.7 λ_2_ = 0.7**	**Chandelier dataset**					**Dining room dataset**				
	**ACC**	**AUC**	**SEN**	**PREC**	**F1**	**ACC**	**AUC**	**SEN**	**PREC**	**F1**
λ_3_ = 0.01	93.78	97.99	91.34	84.75	87.84	92.79	97.48	93.05	77.71	83.77
λ_3_ = 0.05	93.65	98.69	95.27	82.20	88.19	92.55	98.31	93.62	79.04	85.60
λ_3_ = 0.1	94.30	98.76	98.30	87.26	89.16	94.47	98.97	95.10	85.81	88.00
λ_3_ = 0.15	92.49	98.54	94.36	84.20	85.98	93.75	98.21	91.00	80.25	85.94
λ_3_ = 0.2	90.93	97.60	88.98	83.40	82.55	90.38	97.57	90.12	77.27	82.16
λ_1_ = 0.7 λ_3_ = 0.1	**Chandelier dataset**					**Dining room dataset**				
	**ACC**	**AUC**	**SEN**	**PREC**	**F1**	**ACC**	**AUC**	**SEN**	**PREC**	**F1**
λ_2_ = 0.5	91.32	97.98	96.09	85.73	84.59	91.35	98.12	94.67	75.36	83.19
λ_2_ = 0.6	92.75	98.13	93.31	81.02	86.61	91.59	98.95	93.46	77.53	84.68
λ_2_ = 0.7	94.30	98.76	98.30	87.26	89.16	94.47	98.97	95.10	85.81	88.00
λ_2_ = 0.8	92.10	98.44	96.71	87.45	85.84	90.87	98.92	94.43	76.20	81.78
λ_2_ = 0.9	93.52	98.35	94.36	82.52	87.91	92.07	98.31	94.67	75.32	83.72
λ_2_ = 0.7 λ_3_ = 0.1	**Chandelier dataset**					**Dining room dataset**				
	**ACC**	**AUC**	**SEN**	**PREC**	**F1**	**ACC**	**AUC**	**SEN**	**PREC**	**F1**
λ_1_ = 0.5	90.67	97.56	90.78	84.96	83.02	91.59	97.26	87.76	74.63	82.34
λ_1_ = 0.6	94.04	97.87	93.44	87.67	88.95	91.83	98.86	94.34	78.64	84.54
λ_1_ = 0.7	94.30	98.76	98.30	87.26	89.16	94.47	98.97	95.10	85.81	88.00
λ_1_ = 0.8	93.39	98.26	92.23	83.61	87.69	91.11	98.56	94.45	78.88	81.36
λ_1_ = 0.9	92.62	98.12	93.65	81.10	86.71	84.62	98.31	93.83	75.83	76.77

## 5 Conclusions

We develop a weight-aware semi-supervised self-ensembling framework, termed WSSL, to enhance interior decoration style classification by selectively leveraging reliable unlabeled data through an adaptive weighting mechanism. WSSL follows the Mean Teacher paradigm and identifies reliable unlabeled data using a truncated Gaussian function during training. Additionally, WSSL incorporates three novel regularization modules: weighted consistency regularization to enforce consistent predictions for reliable unlabeled data under different perturbations, weighted relation consistency regularization to encourage the consistency of semantic relationships among reliable unlabeled data under different perturbations, and weighted class-aware contrastive learning regularization to enhance the model's discriminative feature learning ability. The synergistic learning of the proposed three regularizations effectively mitigates confirmation bias induced by unreliable pseudo-labels. Extensive experiments on multiple interior decoration style image datasets demonstrate that WSSL consistently outperforms state-of-the-art semi-supervised methods.

Despite the promising performance of WSSL, three is still room for further improvement. For instance, WSSL does not explicitly address domain shifts across different interior decoration style image datasets, which may reduce its generalization ability on unseen styles. In addition, the adaptive weighting function assumes that the maximum values of network predictions follow a Gaussian distribution, which may not always hold and could impact model performance if violated. Furthermore, the current WSSL does not explicitly count for class imbalance, which could be addressed in future work by incorporating imbalance-aware strategies. In future work, we plan to further investigate the generalizability and effectiveness of WSSL in more complex real-world classification tasks, including medical imaging, autonomous driving, and remote sensing.

## Data Availability

The raw data supporting the conclusions of this article will be made available by the authors, without undue reservation.
